# Supplementary material for: Synthesis of a Magnetic Nanostructured Composite Sorbent Only from Waste Materials
Source: Materials (Basel). 2023 Dec 18;16(24):7696. doi: 10.3390/ma16247696 (PMC10744448; doi:10.3390/ma16247696)
Supplement: Supplementary file 1 [file materials-16-07696-s001.zip › materials-2748070-supplementary.pdf]

materials

Supporting information for

Synthesis of a Magnetic Nanostructured Composite Sorbent Only from Waste Materials

Preparation of sorption solutions for pH dependent sorption studies:

Solutions of citric acid (CA, 0.1 M) and disodium hydrogen phosphate (phosphate, 0.2 M) were prepared. According to table S1, the respective solutions were mixed and diluted to 50 mL. The buffer solution (19.5 mL) was then mixed with 0.5 mL of the CV stock solution to prepare the respective solution for the adsorption tests.

Table S1. volumes of stock solutions of CA and phosphate used for the respective buffer solutions.

pH	V_{CA} [ml]	Vphosphate [ml]
3	15.59	4.11
4	12.29	7.71
5	9.7	10.3
6	7.37	12.63
7	3.53	16.47
8	0.53	19.45

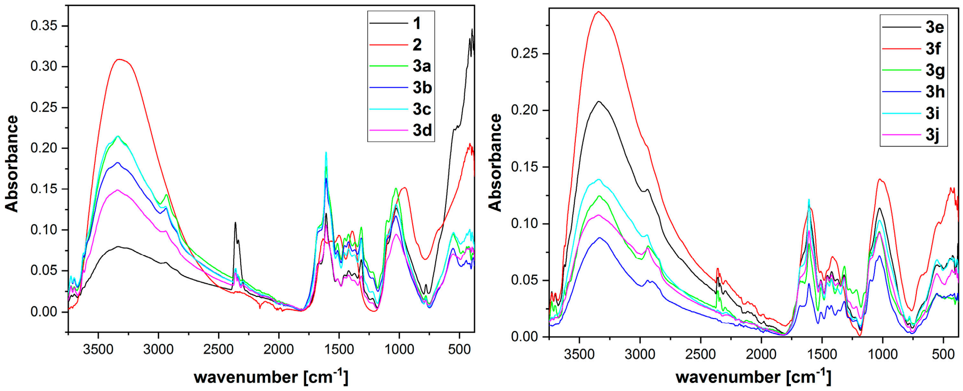

Figure S1. IR spectra of **1**, **2** and **3** (overlayed form).

Kinetics:

If a specific adsorption mechanism can be described using pseudo-first order kinetics, a plot of sorption q_t vs time t should conform to the following formula:

$$q_t = q_e * (1- e^{-k_1 * t}) \quad (S1)$$

with q_t the specific sorption capacity at a certain time t, q_e the equilibrium sorption capacity, k_1 the rate constant and t the time. This formula can also be linearized to

$$\ln(q_t - q_e) = -k_1 * t + \ln(q_e), \quad \text{(S2)}$$

however, in our case it was not necessary to use the linearized plot.

For second-order kinetics, the formula is

$$q_t = k_2 * q_e \hat{} 2 * t / (1 + k_2 * q_e * t) \quad \text{(S3)}$$

with k_2 the pseudo second order rate constant, which can be linearized to

$$t/q_t = 1/(k_2 * q_e \hat{} 2) + t/q_e. \quad \text{(S4)}$$

Also in this case only the nonlinear form was used.

The intraparticle diffusion model assumes that the sorption capacity q_t is proportional to the square root of time, according to

$$q_t = k_{ID} * t^{0.5}, \quad \text{(S5)}$$

with k_{ID} the rate constant for the intraparticle diffusion model. Aside from this formula, there are two other variants of this model.[1] The second model adds a constant c to the equation, according to

$$q_t = k_{ID} * t^{0.5} + c \quad \text{(S6)}$$

This model assumes that there are two sorption processes involved at different times. The third model assumes there are three different intraparticle diffusion processes involved: external surface adsorption, intraparticle diffusion controlled adsorption, and near equilibrium adsorption where the pollutant moves from larger to micropores. In this case q_t needs to be plotted vs $t^{0.5}$ and the plot divided into three linear areas.

A plot of q_t vs t was done and the four functions for pseudo-first order, pseudo-second order and two intraparticle diffusion kinetics were fitted using Origin software, with the orthogonal distance regression mode used for iteration. The reason this iteration mode was used was that due to the sample being left for some time on a magnet to remove the sorbent from the suspension, we feel that there could also be errors on the time axis, and not only on the axis displaying the sorption rate, and the fitting process needs to take this into account as well.

Additionally, for the intraparticle diffusion model a plot was done for q_t vs $t^{0.5}$ and multiple linear fits made for different sections of the plot (Figure S1).

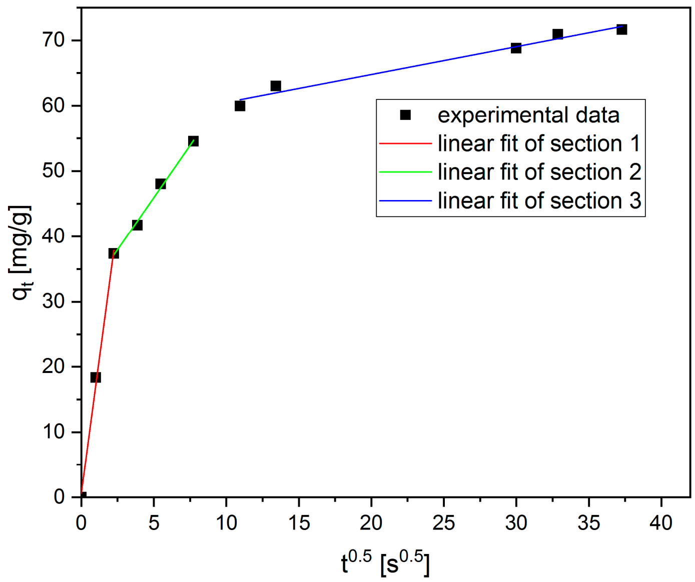

Figure S2. Plot of q_t vs $t^{0.5}$ for the intraparticle diffusion model.

Isotherms:

The Langmuir and Freundlich models were used to attempt to describe the obtained data points. The Langmuir model can be described using the formula

$$q_e = q_{max}*K_L*C_e / (1+K_L*C_e) \quad \text{(S7)}$$

with q_e the equilibrium sorption capacity for a specific equilibrium concentration C_e, q_{max} the maximum equilibrium sorption capacity, and K_L the Langmuir constant. Also this formula can be transformed into a linear form,

$$C_e/q_e = 1/q_{max} * C_e + 1/(K_L*C_e) \quad \text{(S8)}$$

however, in this case the fitting was done only using the nonlinear form.

The Freundlich model is described by

$$q_e = K_F*C_e\text{^}(1/n) \quad \text{(S9)}$$

with K_F the Freundlich constant and n the Freundlich exponential coefficient. The linear form would be

$$\log(q_e) = \log(K_F) + 1/n * \log(C_e). \quad \text{(S10)}$$

A plot of q_e vs C_e was done and the nonlinear functions for Langmuir and Freundlich isotherms fitted using Origin software using the orthogonal distance regression mode for iteration. The reason that this mode was chosen here was because both q_e and C_e depend on the same measurement, and thus would be subject to the same errors.

Thermodynamic parameters:

To determine the thermodynamic parameters ΔG° (Gibbs free energy change), ΔS° (entropy change) and ΔH° (enthalpy change) of the sorption process several formulas need to be combined and used. The van`t Hoff equation states that

$$\Delta G° = -R*T*\ln(K°) \qquad (S11)$$

with R the ideal gas constant, T the temperature, and K° the equilibrium constant for the sorption process. At the same time, the Gibbs free energy can be also calculated according to the Gibbs-Helmhotz equation

$$\Delta G° = \Delta H° - T*\Delta S° \qquad (S12)$$

and the equilibrium constant as

$$K° = q_e / C_e` * C_e°/q_e° \qquad (S13)$$

with C_e` the equilibrium concentration of free pollutant (as mol/L), $C_e°$ = 1 mol/L and $q_e°$ = 1 mol/kg.[2] From equations S9 and S10 one gets

$$\ln(K°) = \Delta S°/R - \Delta H°/(R*T). \qquad (S14)$$

Thus through plotting ln(K°) against 1/T it is possible to calculate ΔH° and ΔS° for the sorption process.

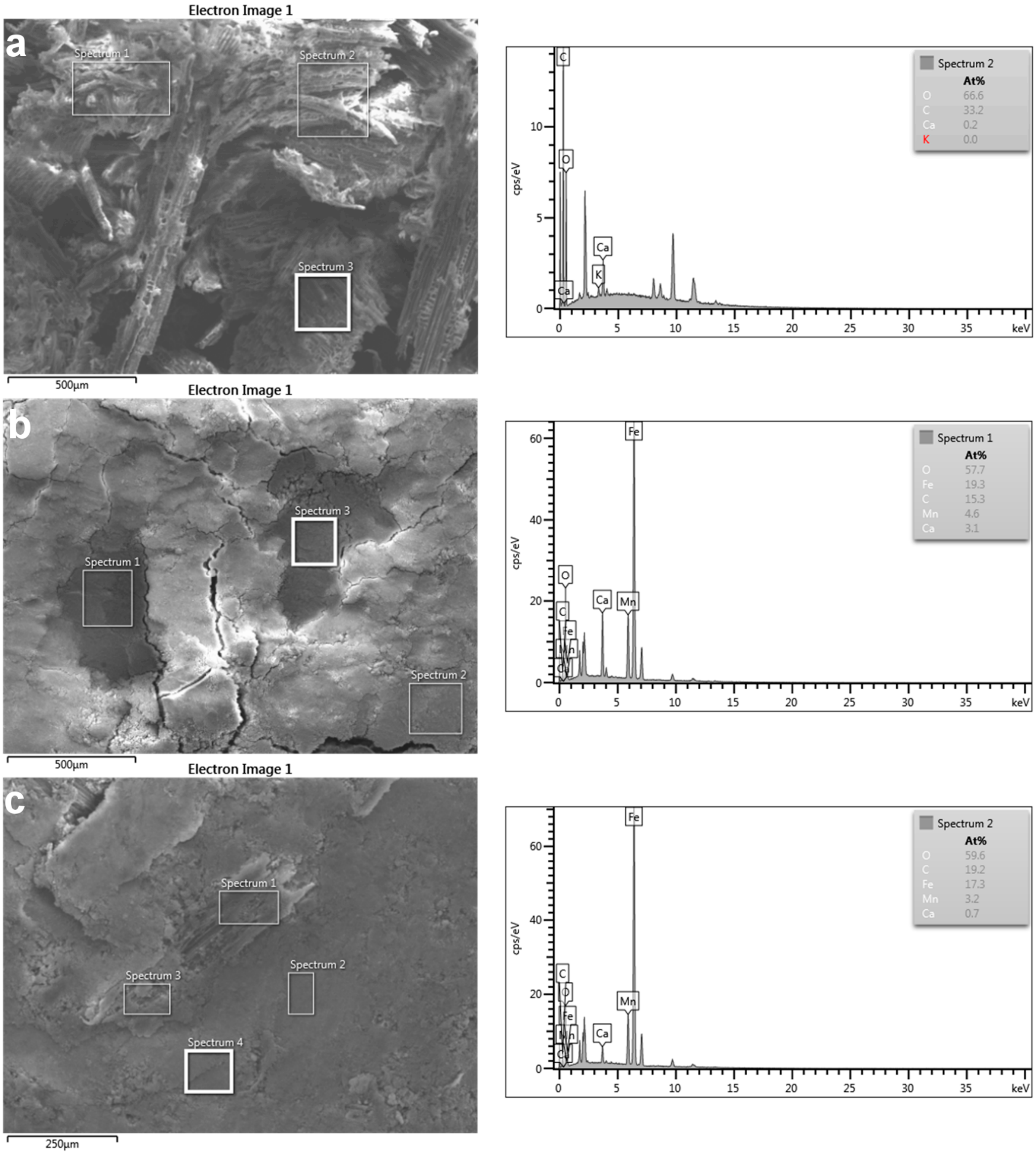

Figure S3. EDX of a) sawdust **1**, b) iron mud **2**, c)composite **3h**.

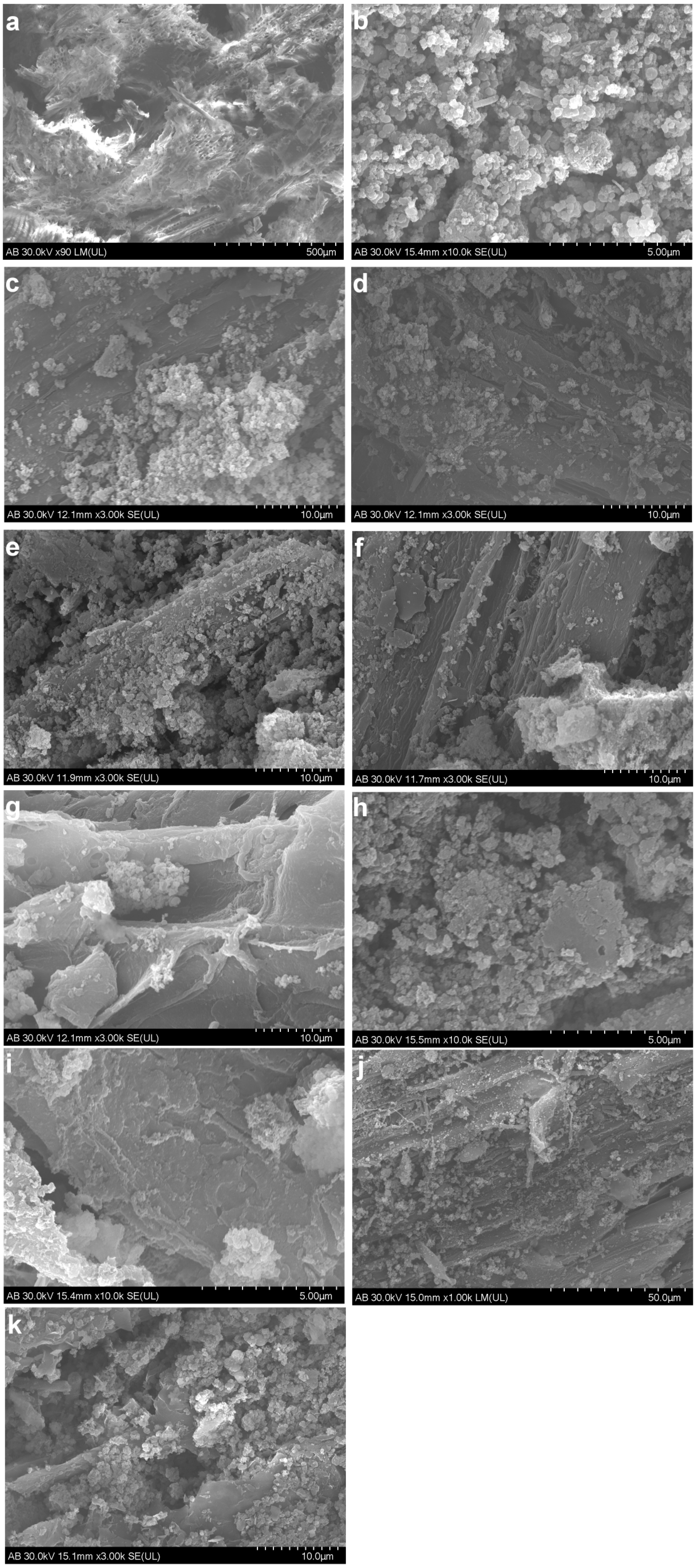

Figure S4. SEM of samples a) **1**; b) **2**; c) **3a**; d) **3b**; e) **3c**; f) **3e**; g) **3f**; h) **3g**; i) **3h**; j) **3i**; k) **3j**.

Table S2. ratios Mn/Fe and Ca/Fe of samples **2**, **3d** and **3h** determined by EDX/XPS.

sample	method	ratio Mn/Fe	ratio Ca/Fe
**2**	EDX	0.24	0.16
**3d**	EDX	0.14	0.03
**3h**	EDX	0.18	0.04
**3d**	XPS	0.28	0.17

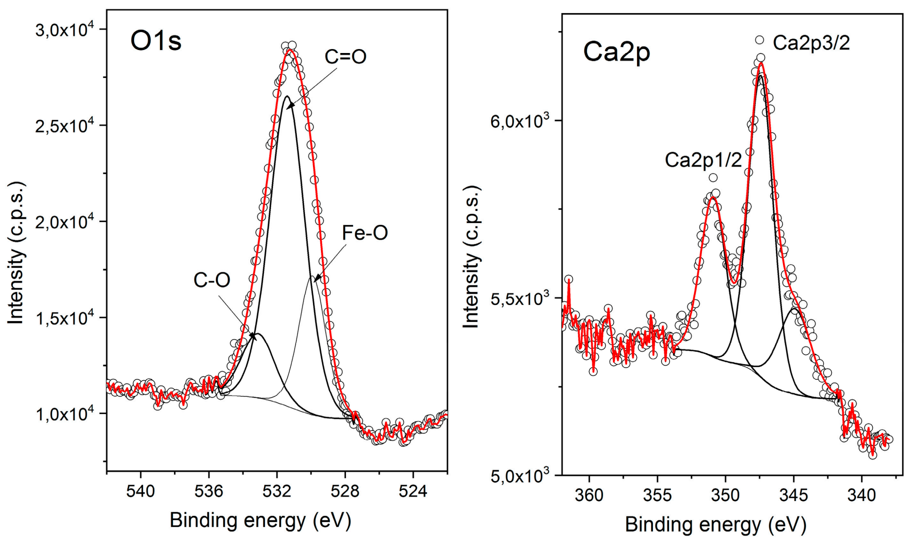

Figure S5. XPS O1s (left) and Ca2p (right) core level spectra of sample **3d**.

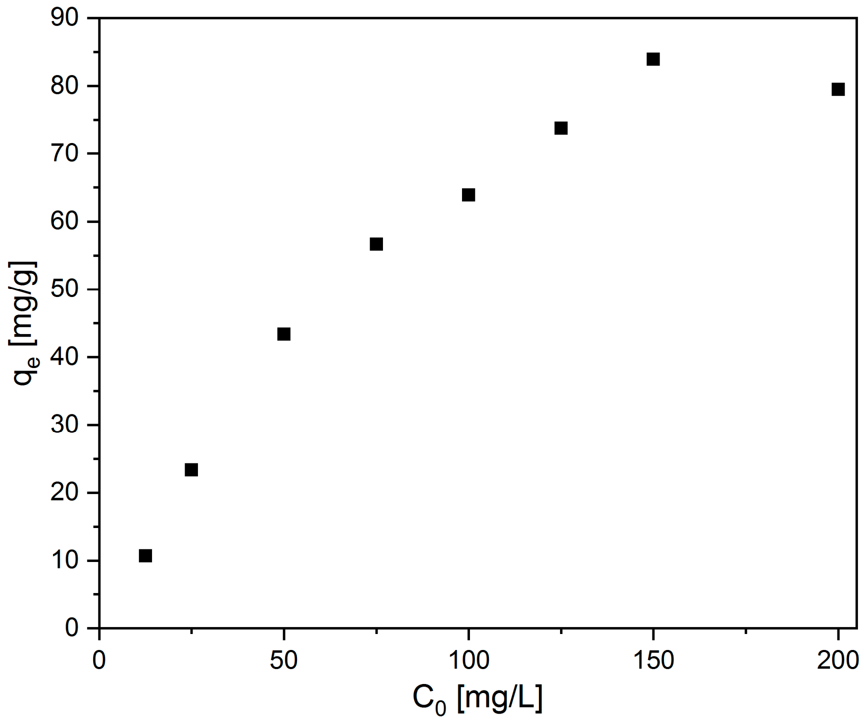

Figure S6. Plot of sorption capacity depending on initial concentration.

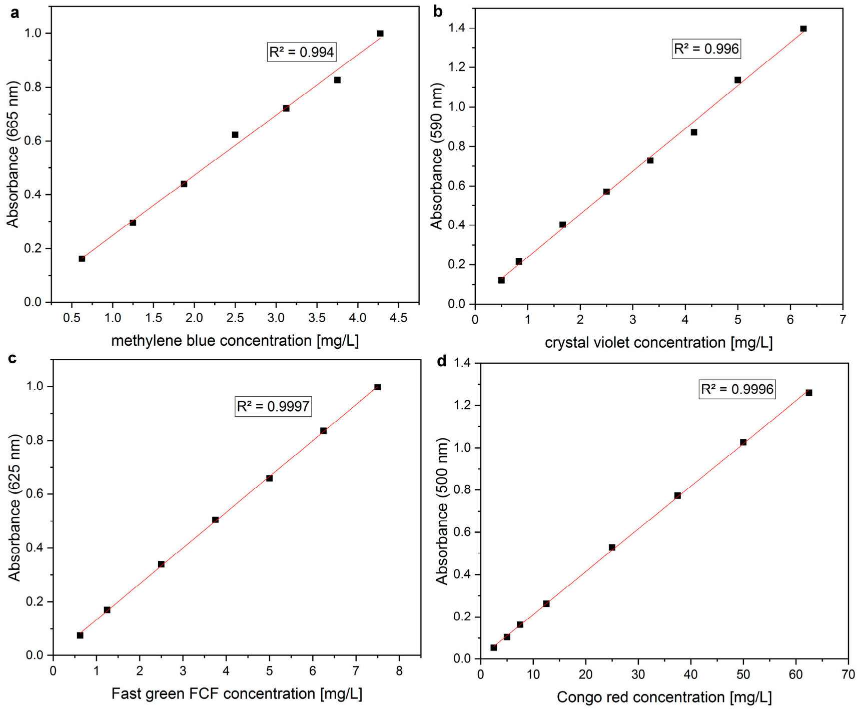

Figure S7. Calibration curves for a) methylene blue, b) crystal violet, c) fast green FCF and d) congo red

References

1. Wu, F.-C.; Tseng, R.-L.; Juang, R.-S. Initial behavior of intraparticle diffusion model used in the description of adsorption kinetics. *Chemical Engineering Journal* **2009**, *153*, 1-8, doi:https://doi.org/10.1016/j.cej.2009.04.042.
2. Chen, T.; Da, T.; Ma, Y. Reasonable calculation of the thermodynamic parameters from adsorption equilibrium constant. *Journal of Molecular Liquids* **2021**, *322*, 114980, doi:https://doi.org/10.1016/j.molliq.2020.114980.
